# How to Prepare an Annual Report

**Published:** 1915-12-11

**Authors:** 


### HOW TO PREPARE AN ANNUAL REPORT.

### A Secretary Gives his View.

I take it that if a secretary is regarded as knowing his business he is allowed by his board to prepare the first rough draft of the annual report. It is left to the secretary to record exactly what has happened during the twelve months. He is entrusted with the setting down of the events of the year, and he writes at length, or briefly, according to the relative importance of the happenings. But it is often the case that the mind of the record-keeper is not the mind of the polished and discreet chronicler, and, for that reason among others, it is well that the annual report should be the product of more than one brain and hand.

A man who is an expert appeal writer may prove a failure at annual report work. Why? Well, for a score of good and sufficient reasons. In an appeal there must be some poetic licence, but there is small room for poetic licence in an annual report. The writing of an annual report is not the most difficult of a good secretary's duties, because all the knowledge necessary may be acquired, whereas appeal writing is a gift. An annual report should be a nest of facts, a "plate of meat," a downright straightforward statement of affairs recently experienced and over. Those who have lived through the affairs recorded are familiar with much that led up to them, but that is not an excuse for making the annual report unintelligible to those who may be regarded as outsiders because they know nothing of the inner circumstances.

### An Appropriate Introduction.

The opening lines of an annual report should prepare the mind of the reader for what is to follow. It is stupid to open brightly upon a very sad tale. Singing and groaning within the covers of one slender pamphlet are absurd. Let success or non-success be written early in the tale that is to be told. Then give reasons for the gain, or the loss. Then follow on with what has been done. Neither gloat too much over the one nor mourn too much over the other. Be brief whenever it is possible without forfeiting an opportunity or missing a chance that may not come another time. Be informing always, but never dull. Try and make the annual report a good piece of work, even when the year does not inspire or warrant praise.

There is much room for difference of honest opinion in the preparation of a hospital's annual report. It is not necessary to record the death of a member of the board very early in the yearly story. The deceased member may have been a most worthy man and an industrious worker in a good cause, but his colleagues who are left behind to carry on the work are not justified in thinking that the majority of the governors will regard the death of one who was personally unknown to them with the same deep concern. The deceased was, as it were, one of the family, and the family should be modest, even about its departed member and his work and memory. The position of the hospital, more particularly all that bears on its future, is more important than condolences, however well earned and however graceful. Besides, deaths, when recorded in annual reports, are stale news as well as sad news.

### What to Avoid.

The work of the salaried members of the hospital staff should never be too fully acknowledged in the pages of any annual report. In these days we all ought to know what are the main duties of each responsible officer, and no business man needs to be told that So-and-so did his or her work very well. It occurs to everybody whose view is practical (and therefore worthy of consideration) that if a lay official is worth retaining, he or she is discharging daily the duties that await attention. The salary list should be the only index necessary in all such matters. A good servant earns a good salary, unless private reasons upset the usual conditions of service.

The balance sheet has passed nowadays out of the hands of the secretary and the cashier into the hands of the chartered accountant. To be sure the accountant is not a conjurer, and he cannot submit a good balance sheet unless he is supplied with creditable material. A satisfactory state of things at the end of the year rests with a group and not with an individual. It may be that the balance sheet is the corner stone of the annual report, yet it is not always difficult to explain an unsatisfactory income and expenditure account. So many circumstances are concerned in the balance sheet. Some hospitals have a knack of obtaining wealth, and some have a way of seeming to have wealth thrust upon them; some are lucky and some are unlucky; some are like books with a good title—they go on and prosper—while others are unfortunately named and appear to be cursed at their birth.

The annual reports of some institutions may be well described as "The Mixture As Before." They are nothing but a weary repetition of previous years' efforts. A few figures are changed and the get-up may be varied, but there are no new thoughts and no promise of coming novelties or departures. In these cases it is not the birth curse, but incompetence which produces the failures. The governors are at fault here, for they should protest against defect or incompetence whenever it reveals itself in the annual report. It is a mistake to expect that the printer will help to make a success of the thing. Printers are not unlike race-horses; they have to be well jockeyed. A secretary and his chief clerk, with the help of a couple of businesslike members of the board, should be able to produce a telling annual report after due and conscientious labour. Where the spirit of personal service is continually present, there the annual report and appeals yield the fullest interest and produce the most successful results.

